# Rettungsdienst, kassenärztlicher Notdienst oder Notaufnahme: Es gelingt der Bevölkerung nur unzureichend, die richtige Ressource für medizinische Akutfälle zu wählen

**DOI:** 10.1007/s00063-021-00820-5

**Published:** 2021-04-20

**Authors:** Bibiana Metelmann, Peter Brinkrolf, Marian Kliche, Marcus Vollmer, Klaus Hahnenkamp, Camilla Metelmann

**Affiliations:** 1 Klinik für Anästhesiologie, Anästhesie, Intensiv‑, Notfall- und Schmerzmedizin, Universitätsmedizin Greifswald, Ferdinand-Sauerbruch-Straße, 17475 Greifswald, Deutschland; 2 Institut für Bioinformatik, Universitätsmedizin Greifswald, Greifswald, Deutschland

**Keywords:** Notarzt, Ärztlicher Bereitschaftsdienst, Notfall, Gemeinsames Notrufleitsystem, Telefonumfrage, Prehospital emergency medicine, First aid, Emergency, Emergency number, Telephone survey

## Abstract

**Hintergrund:**

Bei medizinischen Akutfällen entscheiden Patienten eigenständig, ob sie den Rettungsdienst bzw. den ärztlichen Bereitschaftsdienst der kassenärztlichen Vereinigung anrufen oder sich in der Notaufnahme vorstellen.

**Fragestellung:**

Gelingt der Bevölkerung die angemessene Zuordnung verschiedener dringlicher Erkrankungen zu den unterschiedlichen Systemen?

**Material und Methoden:**

In einer deutschlandweiten, anonymen telefonischen Befragung nach dem Gabler-Häder-Design im Sommer 2018 wurden 708 Personen jeweils 6 verschiedene Szenarien mit medizinischen Akutfällen geschildert. Die Befragten wurden gebeten anzugeben, ob sie kurzfristige medizinische Hilfe für erforderlich hielten. Zusätzlich wurde die subjektive Dringlichkeit der einzelnen Szenarien sowie die Kenntnis der Telefonnummern von Rettungsdienst und ärztlichem Bereitschaftsdienst erhoben.

**Ergebnisse:**

Die Dringlichkeit der Szenarien wurde häufig fehleingeschätzt: bei Szenarien hoher Dringlichkeit zu 20 %, bei mittlerer Dringlichkeit zu 50 % und bei leichter Dringlichkeit zu 27 %. Zusätzlich misslang einigen Befragten die Ressourcenwahl, wenn sie medizinische Hilfe für erforderlich hielten: 25 % würden bei einem Apoplex bzw. Myokardinfarkt keinen Rettungsdienst rufen. Bei Erkrankungen mittlerer Dringlichkeit würden mehr Befragte eigenständig in die Notaufnahme gehen (38 %), als den ärztlichen Bereitschaftsdienst zu alarmieren (46 %).

**Diskussion:**

Das Wissen der Bevölkerung über die verschiedenen Ressourcen bei medizinischen Akutfällen und die Fähigkeit, die Dringlichkeit adäquat einzuschätzen, scheint nicht ausreichend zu sein. Die Lösung könnte neben einer Steigerung der Gesundheitskompetenz eine gemeinsame Telefonnummer für Rettungsdienst und ärztlichen Bereitschaftsdienst mit einheitlichem Abfragetool und Ressourcenzuordnung sein.

**Zusatzmaterial online:**

Zusätzliche Informationen sind in der Onlineversion dieses Artikels (10.1007/s00063-021-00820-5) verfügbar. Sie enthält den Studienfragebogen. Beitrag und Zusatzmaterial stehen Ihnen auf www.springermedizin.de zur Verfügung. Bitte geben Sie dort den Beitragstitel in die Suche ein, das Zusatzmaterial finden Sie beim Beitrag unter „Ergänzende Inhalte“.

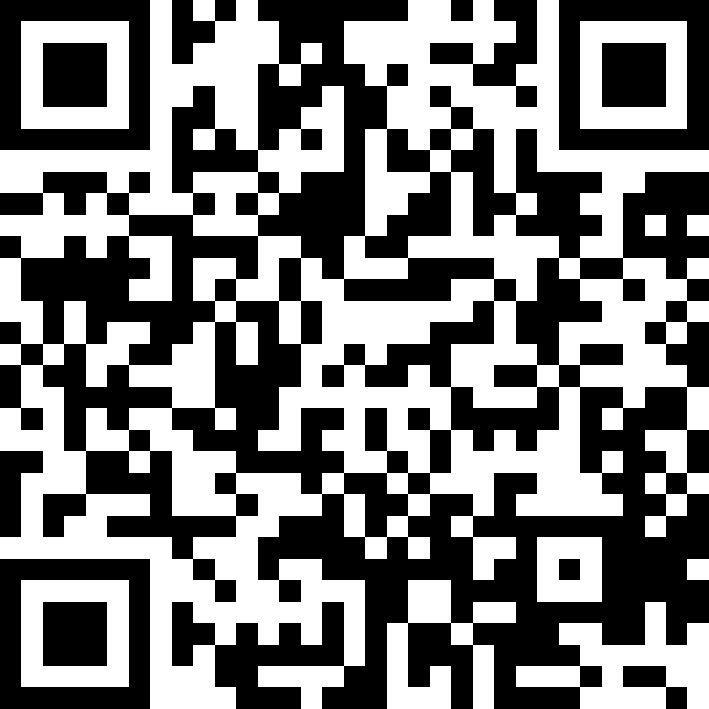

## Abstract (en)

**Background:**

In medical events, patients have to independently decide whom to contact: emergency medical services, medical on-call service or emergency department.

**Objectives:**

Are Germans able to assess the urgency of medical events and choose the correct resource?

**Materials and methods:**

In 2018 a nationwide anonymous telephone survey was done in Gabler–Haeder design. In all, 708 interviewees were presented with six medical scenarios. Participants were asked to rate urgency and to assess whether medical help was necessary within minutes to hours. Telephone numbers of emergency medical services and medical on-call service were inquired.

**Results:**

Urgency of different scenarios was often misjudged: in cases with high, medium, and low urgency the misjudgement rate were 20, 50, and 27%, respectively. If medical help was rated as necessary, some participants chose the wrong service: 25% would not call an ambulance in stroke or myocardial infarction. In cases with medium urgency, more respondents chose to consult an emergency department (38%) than to call medical on-call service (46%).

**Conclusions:**

Knowledge regarding different options for treatment of medical events and competence to assess urgency seem to be too low. Beside efforts to increase health literacy, one solution might be to introduce a joint telephone number for emergency medical services and medical on-call service with a uniform assessment tool and appropriate allocation.

## Hintergrund und Fragestellung

In der Versorgung von medizinischen Akutfällen wird die sektorale Trennung des deutschen Gesundheitssystems besonders deutlich. Derzeit existieren 3 Systeme parallel: Rettungsdienst, ärztlicher Bereitschaftsdienst (ÄBD) der Kassenärztlichen Vereinigung und Notaufnahmen an Krankenhäusern [1, 14, 27]. Diese Systeme sind als gegenseitige Ergänzung konzipiert, werden allerdings unabhängig voneinander organisiert [8, 9, 14, 27]. Die Zuordnung erfolgt momentan durch den Patienten eigenständig und nicht anhand einer Einschätzung durch medizinisches Personal [1, 4].

Die Bevölkerung muss ausreichend über diese 3 Systeme informiert sein, um bei der Auswahl der notwendigen Ressource nicht nur subjektiven Präferenzen oder emotionalen Aspekten zu folgen [5, 10, 25, 30]. Nur so können die Patienten bei medizinischen Problemen kenntnisbasiert das für diesen Fall adäquate System wählen und kontaktieren.

Hieraus ergab sich die Frage, ob das Wissen über diese Systeme in der Bevölkerung ausreichend hoch vorhanden ist und ob die Bevölkerung die Dringlichkeit verschiedener Situationen adäquat einschätzen kann.

## Studiendesign und Untersuchungsmethoden

Im Rahmen dieser Querschnittsstudie wurde entsprechend den Richtlinien der GESIS, Leibniz-Institut für Sozialwissenschaften, eine telefonische Befragung einer Stichprobe der deutschen Bevölkerung durchgeführt [12]. Hierzu wurden vom 11. Juni 2018 bis zum 26. August 2018 von Montag bis Freitag von 9.00 bis 21.00 Uhr und Samstag von 10.00 bis 20.00 Uhr mit einer Dual-Frame-Stichprobe in gleichem Anteil Festnetz- und Mobilfunknummern, die nach dem Gabler-Häder-Design generiert waren, über „random digit dialing“ angerufen [11]. Dabei wurden bis zu 8 Kontaktversuche zum Erreichen eines Anschlusses unternommen und Telefonnummern in 100er-Blöcken genutzt [11]. Ausgeschlossen wurden nichtvergebene und geschäftliche Rufnummern. Eingeschlossen in die Befragung wurden Personen, die der Befragung zustimmten, nach eigenen Angaben mindestens 16 Jahre alt waren, fließend Deutsch sprachen und ihren Hauptwohnsitz in Deutschland hatten. Zur standardisierten Befragung wurden alle Telefonate anhand eines Fragebogens durch nur einen Untersucher durchgeführt.

Um die Kenntnisse und das Vorgehen bei medizinischen Akutsituationen praxisnah zu prüfen, wurden während des Telefonats in randomisierter Reihenfolge 6 fiktive Szenarien mit medizinischen Akutsituationen als Tonbandaufnahme eingespielt. Dabei waren jeweils 2 Szenarien so ausgelegt, dass eine Alarmierung des Rettungsdiensts bzw. des kassenärztlichen Notdiensts bzw. ein Abwarten und ggf. eine Vorstellung beim Hausarzt im Verlauf indiziert waren. Die Szenarien wurden durch die Autoren entwickelt und in einem Pretest durch Notärzte, Rettungsdienstmitarbeiter, Leitstellendisponenten und Sprachwissenschaftler validiert. Folgende Szenarien wurden konstruiert: eine Apoplexsymptomatik mit Hemiparese, ein typisches Beschwerdebild eines Myokardinfarkts, eine Lumboischalgiesymptomatik, ein persistierender Harnwegsinfekt mit Schmerzen, ein zufällig gemessener Bluthochdruck ohne Symptome und ein seit 2 Tagen bestehender grippaler Infekt. Die Befragten wurden gebeten, sich in die Rolle eines Patienten zu versetzen, der an einem Samstag diese Situation erlebt. Nach jedem einzelnen Szenario wurden die Teilnehmer zur subjektiven Einschätzung der Situation und zum weiteren Vorgehen befragt. Hierfür wurde erfasst, ob sie kurzfristig (innerhalb der nächsten Minuten bis Stunden) medizinische Hilfe bräuchten und, falls ja, an wen sie sich wenden würden. Außerdem wurde gefragt, für wie akut (1 = nicht akut bis 10 = sehr akut) sie die Situation einschätzten, und dies anschließend in die Kategorien 1 = nicht dringlich, 2–4 = leicht dringlich, 5–7 = mittel dringlich, 8–10 = sehr dringlich umcodiert. Zusätzlich wurde die Bekanntheit der unterschiedlichen Ressourcen bei medizinischen Akutfällen erfasst. Der vollständige Fragebogen inklusive Beschreibung der Szenarien befindet sich im elektronischen Zusatzmaterial online.

Die Fallzahlplanung ergab, aufbauend auf einer Studie von Kirkby, bei einer konservativen Schätzung einer korrekten Beurteilungsrate der Fallszenarien von 50 % eine erforderliche Mindestanzahl von 706 Interviews, um die Spannweite des 95 %-Konfidenzintervalls auf maximal 7,5 % zu begrenzen [13]. Die statistische Analyse erfolgte mit der Software RStudio® (RStudio PBC, Boston, U.S.A.). Ein positives Votum der Ethikkommission der Universitätsmedizin Greifswald (Interne Reg. Nr.: BB 078/18) liegt vor.

## Ergebnisse

Im Rahmen der Befragung wurden 14.000 Telefonnummern angewählt. Es konnten 708 vollständige Interviews realisiert werden. Tab. 1 zeigt die Stichprobenausschöpfung nach Anwendung der Ein- und Ausschlusskriterien.

**Table 1:** 

	Absolut	Relativ (in %)
**1. Insgesamt gewählte Nummern**	14.000	100 % = 14.000
Nummer nicht vergeben	8043	57,5
Geschäftliche Rufnummer	265	1,9
Fax	185	1,3
Summe der im ersten Schritt ausgeschlossenen Nummern	*8493*	*60,7*
**2. Existente und private Nummern**	5507	100 % = 5507
Stets Anrufbeantworter	1776	32,2
Nie abgehoben	1517	27,5
Einschlusskriterien nicht erfüllt	93	1,7
Vereinbarter Termin nicht eingehalten	322	5,8
Aufgelegt vor Erfragung des Einverständnisses	270	4,9
Summe der im zweiten Schritt ausgeschlossenen Nummern	*3978*	*72,2*
**3. Infrage kommende Nummern**	1529	100 % = 1529
Nonresponse – Befragung abgelehnt	806	52,7
Nonresponse – Befragung abgebrochen	15	1,0
Realisierte Interviews	*708*	*46,3*

Von 14.000 Nummern waren 1529 infrage kommende Nummern vorhanden und 708 (46,3 %) Befragungen wurden vollständig durchgeführt

**Figure 1:**
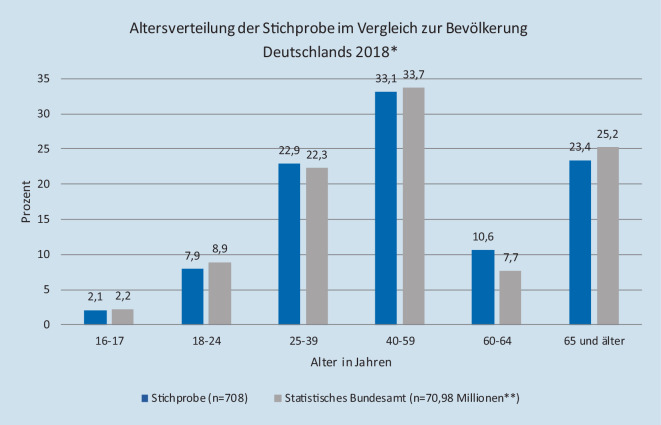

Durch das Gabler-Häder-Design wurde eine repräsentative Stichprobe der deutschen Bevölkerung angestrebt. In der Stichprobe waren Personen aus allen Bundesländern vertreten. Bei der Altersverteilung gab es keinen signifikanten Unterschied zwischen der Telefonstichprobe und der Bevölkerungsstatistik des Statistischen Bundesamts (*p* = 0,099; Abb. 1). Jedoch sind in der Stichprobe mit 57,6 % (*n* = 408) signifikant mehr Frauen als Männer (42,4 %; *n* = 300) vertreten im Vergleich zur deutschen Bevölkerung mit 50,7 % (*n* = 36,54 Mio.; *p* < 0,05). Zusätzlich lebten signifikant mehr Personen der Stichprobe in ländlichen Regionen (unter 4999 Einwohner) mit 26 % (*n* = 184) im Vergleich zu 14 % (*n* = 11,74 Mio.; *p* < 0,05). Der prozentuale Anteil an Personen mit hohem Bildungsabschluss war mit 29 % signifikant höher als in der Bevölkerung mit 13 % (*p* < 0,05).

**Figure 2:**
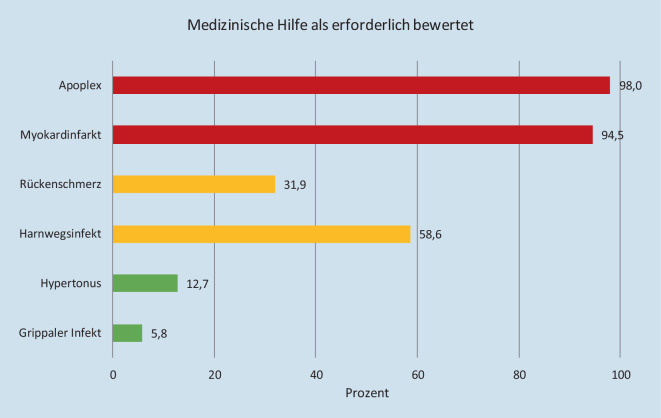

Bei den Szenarien Apoplex und Myokardinfarkt hielten 98,0 % (*n* = 694) bzw. 94,5 % (*n* = 669) der Befragten medizinische Hilfe für nötig. Bei einem Harnwegsinfekt gaben 58,6 % der Befragten an, dass sie kurzfristig medizinische Hilfe benötigen (siehe Abb. 2).

**Figure 3:**
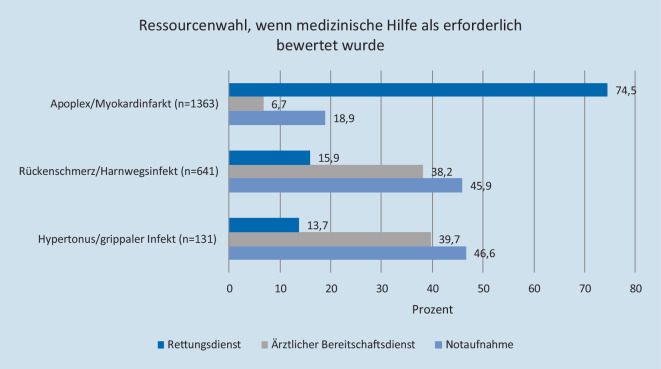

Schätzten die Befragten eine medizinische Hilfe als erforderlich ein, wurden sie gefragt, welche Ressource sie wählen würden. Abb. 3 zeigt, dass bei sehr dringlichen Szenarien 74,5 % (*n* = 1015) den Rettungsdienst alarmieren, jedoch 6,7 % (*n* = 91) den ÄBD alarmieren würden und sich 18,9 % (*n* = 257) selbstständig in der Notaufnahme vorstellen würden. Bei Szenarien mit mittlerer Dringlichkeit würden 38,2 % (*n* = 245) den ÄBD alarmieren, aber 45,9 % (*n* = 294) sich selbstständig in der Notaufnahme vorstellen. Ähnliche Prozentwerte zeigen sich bei Fällen mit leichter Dringlichkeit (39,7 %, *n* = 52 ÄBD und 46,6 %, *n* = 61 Notaufnahme).

**Figure 4:**
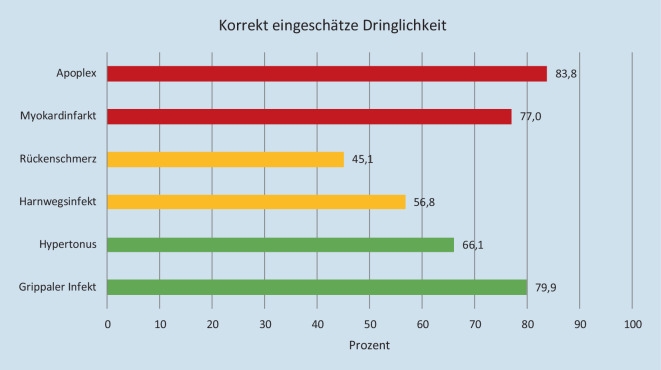

Abb. 4 illustriert die von den Teilnehmern subjektiv eingeschätzte Dringlichkeit in Relation zur tatsächlichen Dringlichkeit. Die korrekte Einschätzung der Dringlichkeit gelang beim Szenario Apoplex 83,8 % (*n* = 593) der Befragten, beim Myokardinfarkt 77 % (*n* = 545), beim Rückenschmerz 45,1 % (*n* = 319), beim Harnwegsinfekt 56,8 % (*n* = 402), beim zufällig entdeckten Hypertonus 66,1 % (*n* = 468) und bei dem grippalen Infekt 79,9 % (*n* = 565) der Befragten.

**Figure 5:**
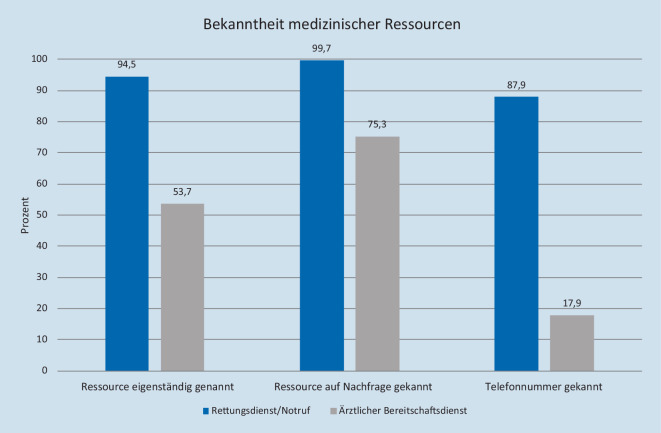

Als Ressourcen, unter welcher man medizinische Hilfe bekommen könnte, nannten 94,5 % (*n* = 669) der Befragten den Rettungsdienst/Notruf eigenständig und 99,7 % (*n* = 706) kannten diesen auf Nachfrage. 87,9 % (*n* = 622) der Befragten konnten die 112 als gültige Telefonnummer nennen (siehe Abb. 5). Den ÄBD nannten 53,7 % (*n* = 380) der Befragten eigenständig und 75,3 % (*n* = 533) kannten diesen auf Nachfrage. Eine gültige Telefonnummer (116117 oder regionale Telefonnummer) nannten 17,9 % (*n* = 127) der Teilnehmer.

## Diskussion

Im deutschen Gesundheitssystem kann ein Patient bei neu aufgetretenen Beschwerden eigenständig entscheiden, welche Gesundheitseinrichtung er kontaktiert: den Rettungsdienst, den ärztlichen Bereitschaftsdienst oder die Notaufnahme [4]. Zusätzlich kann er auch zunächst abwarten und keine medizinische Hilfe anfordern. Um diese Entscheidung eigenständig sicher treffen zu können, muss der Patient ausreichend über diese Ressourcen informiert sein und zusätzlich die Erkrankungsschwere adäquat einschätzen können [25].

Diese Telefonumfrage zeigt, dass 12 % der Befragten die Nummer 112 zur Alarmierung des Rettungsdienstes nicht kannten. Diese Zahl scheint über die letzten 15 Jahre konstant zu bleiben [19].

Der Bevölkerung scheint es schwer zu fallen, die Dringlichkeit der Situation adäquat einzuschätzen. In der Telefonbefragung haben bei der Schilderung typischer Beschwerden eines Apoplex und eines Myokardinfarkts 20 % die Dringlichkeit unterschätzt. Dies deckt sich mit den Ergebnissen einer Telefonbefragung von 2013 [16]. Die Fehleinschätzung der Dringlichkeit kann zu erheblichen Zeitverzögerungen bis zum ersten medizinischen Kontakt und der erforderlichen Therapie führen. In unserer Befragung riefen 25 % der Befragten bei diesen lebensbedrohlichen Szenarien nicht den Rettungsdienst.

Gleichzeitig wird die Dringlichkeit bei nicht-/leicht dringlichen Fälle in circa 30 % überschätzt und bei mitteldringlichen Fällen gelang weniger als der Hälfte der Befragten die richtige Dringlichkeitseinstufung. Ähnliche Ergebnisse zeigen auch Studien zu Notaufnahmepatienten [10, 24, 30, 31]. So schätzten sich 90 % der Patienten, die sich fußläufig in der Notaufnahme vorstellen, als Notfall ein, wobei in 48–68 % der Fälle die Dringlichkeit der Behandlung durch die medizinischen Laien im Vergleich zu Notaufnahmeärzten höher eingeschätzt wurde [20, 30, 31]. Erfahrungen aus der Schweiz zeigen, dass 70 % der Anrufer bei der medizinischen computerassistierten Telefontriage die Dringlichkeit ihrer Beschwerden falsch bewerteten [17]. Und nur bei 2 % der Anrufer, die eine Notaufnahme aufsuchen wollten, war dies tatsächlich indiziert [17].

Obwohl eine Alarmierung des ÄBD indiziert gewesen wäre, würden bei mitteldringlichen Fällen mit 45,9 % mehr Personen eigenständig die Notaufnahme aufsuchen als den ÄBD (38,2 %) zu alarmieren. Und auch bei den leicht bzw. nichtdringlichen Fällen gehen mehr Personen in die Notaufnahme (46,6 %), als dass sie den ÄBD (39,7 %) kontaktieren. Die verminderte Nutzung einer Ressource führt konsekutiv zu einer Belastung der anderen Ressourcen [10, 14, 27, 30]. Sowohl im Rettungsdienst als auch in der Notaufnahme war in den letzten Jahren ein deutlich gesteigertes Patientenaufkommen zu verzeichnen [1, 19, 26, 27, 29]. Diese Mehrbelastungen sind mit erheblichen Kosten verbunden und bergen das Risiko, dass die Kapazitäten für die Behandlungen von lebensbedrohlich erkrankten Personen nicht ausreichen [15, 26].

Neben der Fehleinschätzung der Dringlichkeit könnte die geringe Nutzung der Ressource „ärztlicher Bereitschaftsdienst“ auch darin liegen, dass jeder Vierte auch auf Nachfrage diese Ressource nicht kannte. Und nur 18 % der Befragten konnten eine korrekte Telefonnummer zur Alarmierung des ÄBD nennen. Dies ist eine Steigerung der Bekanntheit im Vergleich zu einer 2013 (ein Jahr nach der Einführung der 116117 [7]) in der Westpfalz durchgeführten Telefonbefragung, bei der nur 2 % die Nummer 116117 kannten [16]. Doch auch 5 Jahre später und nach großen Werbekampagnen ist die Nummer weitgehend unbekannt.

Der Bevölkerung ist mehrheitlich nicht bewusst, welches Einsatzspektrum der Rettungsdienst bzw. der ärztliche Bereitschaftsdienst übernimmt [16, 27]. Im Sinne der Steigerung der Gesundheitskompetenz sollten Maßnahmen ergriffen werden, um das Wissen zu Gesundheit und Krankheit und Handlungskompetenzen in der Bevölkerung zu stärken, damit diese befähigt ist, eigenständig adäquate Entscheidungen zu treffen [3, 28]. Die Schulungsmaßnahmen müssen gleichzeitig die Über- und Unterschätzung adressieren und es besteht das Risiko, dass die Fehleinschätzung nur in eine Richtung verschoben und hier verstärkt wird.

Wichtig scheint, dass neben einem größeren Wissen über die verschiedenen Ressourcen auch ein höheres Bewusstsein für unterschiedliche Dringlichkeiten geschaffen wird [16, 19]. Hierbei muss davon ausgegangen werden, dass der Schulungseffekt erst mit deutlicher Verzögerung eintritt [1, 14, 16] und enorme Anstrengungen erforderlich sind, um die gewünschten Effekte zu erzielen.

Neben der Steigerung der Gesundheitskompetenz ist darüber hinaus eine stärkere Synergie der akutmedizinischen Ressourcen zu fordern. Die eigenständige Zuordnung durch Patienten zu einem der 3 akutmedizinischen Versorgungssysteme wurde als suboptimal identifiziert [1, 4, 10, 14, 19, 20, 27, 30]. Daher hat das Bundesministerium für Gesundheit einen Referentenentwurf vorgelegt, der eine Neugliederung der Notfallversorgung in Deutschland vorsieht [2]. Dieser beinhaltet eine engere Verknüpfung der unterschiedlichen Partner in der Patientenversorgung; diese sektorenübergreifende Zusammenarbeit wird stark diskutiert [10, 27]. Zentrales Element des Maßnahmenbündels ist ein gemeinsames Notfallleitsystem, das von der Bevölkerung bei medizinischen Akutfällen kontaktiert werden kann und die richtige Ressource zuordnet [2].

Die Etablierung einer gemeinsamen Telefonnummer für Rettungsdienst und ÄBD scheint ein Lösungsweg zu sein [1, 10, 16, 18, 21, 27]. Dies wurde in einigen Regionen Deutschlands als Modellprojekt realisiert und ist in vielen Ländern Europas seit Jahren erfolgreich etabliert [10, 14, 17, 22]. Über eine solche Nummer kann nach einer standardisierten Abfrage eine Zuweisung zum geeigneten System erfolgen [1, 10]. Somit könnte man auch Patienten, die die Dringlichkeit der Situation fehleinschätzen, schnell der richtigen Versorgungsform zukommen lassen [1, 10, 21]. Dies würde die Patienten von der Verantwortung entlasten, in einer für sie belastenden Situation diese schwierige Entscheidung treffen zu müssen [27]. Zusätzlich wird eine erheblich geringere Fehlnutzung der einzelnen Ressourcen erwartet. Neben einer gemeinsamen Telefonnummer besteht auch die Möglichkeit, ein gemeinsames Abfragetool mit intelligenter Verknüpfung zu entwickeln, das unabhängig von der gewählten Nummer eine schnelle Zuweisung sicher ermöglicht [27]. Dabei ist es essenziell, dass ein einheitliches Abfragesystem zuvorderst darauf abzielt, zeitkritische Notfälle unmittelbar zu identifizieren, um in diesen Fällen schnellstmöglich den Rettungsdienst zu alarmieren. Derzeit werden mehrere Abfragetools international evaluiert [6, 10, 14, 18, 23].

### Limitationen der Studie

Einschränkend muss beachtet werden, dass das Design einer Telefonumfrage immer nur eine Stichprobe der Bevölkerung darstellt. Durch das Gabler-Häder-Design wurde versucht, die Übertragbarkeit der Studienergebnisse zu erhöhen. Es lässt sich vermuten, dass die Entscheidung der Dringlichkeit und die Ressourcenwahl von verschiedenen Faktoren beeinflusst werden (z. B. sozioökonomischer und kultureller Hintergrund, Vorerkrankungen, soziales Netzwerk und emotionale Faktoren). Es kann nicht ausgeschlossen werden, dass sich die Befragten in einer tatsächlichen Akutsituation anders entscheiden, als sie es nach der telefonischen Schilderung des Szenarios getan haben.

## Fazit

- 88 % der Befragten kennen die Nummer des Rettungsdiensts, aber nur 18 % kennen die Nummer des ärztlichen Bereitschaftsdiensts.
- Die Dringlichkeit einer geschilderten Apoplex- und Myokardinfarktsymptomatik wird von jedem Vierten unterschätzt und 2–5 % der Stichprobe halten hier kurzfristige medizinische Hilfe für nicht erforderlich.
- Bei Erkrankungen mittlerer Dringlichkeit würden mehr Befragte eigenständig in die Notaufnahme gehen, als dass sie den ärztlichen Bereitschaftsdienst alarmieren würden.
- Der Kenntnisstand über die verschiedenen Ressourcen bei medizinischen Akutfällen und die Fähigkeit, die Dringlichkeit dieser Situationen adäquat einzuschätzen, scheint in der deutschen Bevölkerung nicht ausreichend zu sein. Eine Steigerung der Gesundheitskompetenz sollte angestrebt werden.
- Eine geeignete Lösung zur adäquaten Zuordnung der Ressourcen Rettungsdienst und ärztlicher Bereitschaftsdienst scheint eine gemeinsame Koordination mit einheitlichem Abfragetool zu sein.

## Supplementary Information




