# Supplementary material for: Emergency medical service, medical on-call service, or emergency department: Germans unsure whom to contact in acute medical events
Source: Med Klin Intensivmed Notfmed. 2021 Apr 20;117(2):144–51. [Article in German] doi: 10.1007/s00063-021-00820-5 (PMC8897349; doi:10.1007/s00063-021-00820-5)
Supplement: Supplementary file 1 [file 63_2021_820_MOESM1_ESM.pdf]

Dringliches medizinisches Hilfeersuchen der Bevölkerung: Gelingt es der deutschen Bevölkerung einzuschätzen, welche Ressource erforderlich ist?

Fragebogen

Studienleiter:

- Dr. med. Peter Brinkrolf, Oberarzt in der Klinik für Anästhesiologie, Universitätsmedizin Greifswald; Stellvertretender Ärztlicher Leiter Rettungsdienst Landkreis Vorpommern-Greifswald

Beteiligte Ärzte:

- Prof. Dr. med. Klaus Hahnenkamp, Direktor der Klinik für Anästhesiologie, Universitätsmedizin Greifswald
- Dr. med. Bibiana Metelmann, Assistenzärztin in der Klinik für Anästhesiologie, Universitätsmedizin Greifswald
- Dr. med. Camilla Metelmann, Assistenzärztin in der Klinik für Anästhesiologie, Universitätsmedizin Greifswald
- Marian Kliche, Student der Humanmedizin, Universität Greifswald

Fragebogen

Inhaltsverzeichnis

1 Gesprächsbeginn – Einverständnis der Befragten einholen 3
2 Fragen zu den verschiedenen medizinischen Erkrankungen 3
2.1 Rettungsdienst / Notruf (112) 3
2.1.1 Apoplex 3
2.1.2 Myokardinfarkt 3
2.2 Ärztlicher Bereitschaftsdienst (116117) 3
2.2.1 Rückenschmerz 3
2.2.2 Harnwegsinfekt 3
2.3 Abwarten / Hausarzt in den nächsten Tagen vorstellen 4
2.3.1 Hypertonus 4
2.3.2 Grippaler Infekt 4
3 Allgemeine Daten für die statistischen Einordung 4
3.1 Bekanntheit von medizinisch mobilen Ressourcen 4
3.2 Angaben zum Befragten 4
3.3 Medizinische Vorkenntnisse 5
4 Abschluss 5

Fragebogen

1 Gesprächsbeginn – Einverständnis der Befragten einholen

„Guten Tag. Mein Name ist Marian Kliche von der Universitätsmedizin Greifswald. Wir führen eine wissenschaftliche Befragung zur medizinischen Versorgung durch. Die Teilnahme ist freiwillig, aber es ist sehr wichtig, dass möglichst alle ausgewählten Personen teilnehmen, damit die Umfrage ein richtiges Ergebnis liefert. Die Auswertung erfolgt selbstverständlich anonym. Die Befragung wird circa neun Minuten dauern.

Sind Sie mit einer Befragung einverstanden?“ ☐ ja ☐ nein

„Voraussetzung für das Interview ist, dass Sie mindestens 16 Jahre alt sind und fließend deutsch sprechen. Trifft dies auf Sie zu?“ ☐ ja ☐ nein, unter 16 >> „Kann ich Deine Eltern sprechen?“
☐ nein, nicht fließend deutsch

2 Fragen zu den verschiedenen medizinischen Erkrankungen

„Als erstes möchte ich Ihnen 6 Situationen mit medizinischen Fällen schildern und Sie antworten mir bitte, was Sie tun würden. Zudem werde ich Sie nach jedem Fallbeispiel bitten zu sagen, wie akut und wie emotional belastend der Fall ist. Für die Bewertung nutzen Sie bitte die Skala 1-10. Die Zahl 1 bedeutet nicht akut bzw. nicht belastend und die Zahl 10 sehr akut bzw. sehr belastend.
Dann beginnen wir mit dem ersten Fallbeispiel.“
Reihenfolge innerhalb dieses Blockes wird bei jedem Anruf durch Zufall festgelegt.

2.1 Rettungsdienst / Notruf (112)

2.1.1 Apoplex

„Es ist Samstag Nachmittag und Sie haben Besuch. Plötzlich bemerken Sie, dass Sie die rechte Hand nicht mehr anheben und bewegen können. Als Sie dies Ihrem Besuch mitteilen wollen, hört sich Ihre Stimme verschwommen und undeutlich an.“
„Brauchen Sie kurzfristig, also in den nächsten Minuten bis Stunden, medizinische Hilfe?“
☐ *„nein, was machen Sie als nächstes?“* ______ ☐ *„ja, wen kontaktieren Sie?“* ______
„Wie akut ist der geschilderte Fall? 1=nicht akut, 10=sehr akut“ ______
„Wie emotional belastend ist der geschilderte Fall? 1 =nicht belastend, 10 = sehr belastend“ ______

2.1.2 Myokardinfarkt

„Sie kommen vom Wochenendeinkaufen am Samstag zurück und tragen schwere Einkaufstüten. Sie entwickeln zunehmende Schmerzen in der Brust, die in den Unterkiefer und den linken Arm ziehen und es fühlt sich so an, als würde ein Elefant auf Ihrer Brust sitzen. Sie werden von Passanten gefragt, ob Sie Hilfe bräuchten, da Sie sehr blass und verschwitzt seien.“
„Brauchen Sie kurzfristig, also in den nächsten Minuten bis Stunden, medizinische Hilfe?“
☐ *„nein, was machen Sie als nächstes?“* ______ ☐ *„ja, wen kontaktieren Sie?“* ______
„Wie akut ist der geschilderte Fall? 1=nicht akut, 10=sehr akut“ ______
„Wie emotional belastend ist der geschilderte Fall? 1 =nicht belastend, 10 = sehr belastend“ ______

2.2 Ärztlicher Bereitschaftsdienst (116117)

2.2.1 Rückenschmerz

„Sie haben Ihre Möbel im Wohnzimmer am Samstag umgestellt. Nach dem arbeitsintensiven Tag sind Sie erschöpft und gehen schlafen. Am nächsten Morgen haben Sie starke Rückenschmerzen, die in das Bein ziehen. Das Bein fühlt sich leicht kribbelig an. Bei Bewegungen verschlimmern sich die Beschwerden.“
„Brauchen Sie kurzfristig, also in den nächsten Minuten bis Stunden, medizinische Hilfe?“
☐ *„nein, was machen Sie als nächstes?“* ______ ☐ *„ja, wen kontaktieren Sie?“* ______
„Wie akut ist der geschilderte Fall? 1=nicht akut, 10=sehr akut“ ______
„Wie emotional belastend ist der geschilderte Fall? 1 =nicht belastend, 10 = sehr belastend“ ______

Fragebogen

2.2.2 Harnwegsinfekt

„Es ist Samstag Mittag. Seit drei Tagen brennt es sehr unangenehm beim Wasserlassen und Sie müssen sehr häufig zur Toilette. Zudem fühlen Sie sich abgeschlagen und haben starke Schmerzen in der Nierengegend.“
„Brauchen Sie kurzfristig, also in den nächsten Minuten bis Stunden, medizinische Hilfe?“
☐ *„nein, was machen Sie als nächstes?“* ☐ *„ja, wen kontaktieren Sie?“*
„Wie akut ist der geschilderte Fall? 1=nicht akut, 10=sehr akut“ ______
„Wie emotional belastend ist der geschilderte Fall? 1 =nicht belastend, 10 = sehr belastend“ ______

2.3 Abwarten / Hausarzt in den nächsten Tagen vorstellen

2.3.1 Hypertonus

„Sie haben ein Blutdruckmessgerät geschenkt bekommen. Sie testen in einem ruhigen Moment das Gerät. Es zeigt an, dass die Werte erhöht sind **(bei Nachfragen: 160/95 mmHg)***. Eine erneute Messung am anderen Arm und auch in den nächsten Tagen ergibt ähnliche Werte. Auch nun am Samstag Mittag sind die Werte erhöht.“*
„Brauchen Sie kurzfristig, also in den nächsten Minuten bis Stunden, medizinische Hilfe?“
☐ *„nein, was machen Sie als nächstes?“* ☐ *„ja, wen kontaktieren Sie?“*
„Wie akut ist der geschilderte Fall? 1=nicht akut, 10=sehr akut“ ______
„Wie emotional belastend ist der geschilderte Fall? 1 =nicht belastend, 10 = sehr belastend“ ______

2.3.2 Grippaler Infekt

„Seit zwei Tagen haben Sie einen grippalen Infekt. Am Samstag Mittag messen Sie eine Körpertemperatur von 38°C.“
„Brauchen Sie kurzfristig, also in den nächsten Minuten bis Stunden, medizinische Hilfe?“
☐ *„nein, was machen Sie als nächstes?“* ☐ *„ja, wen kontaktieren Sie?“*
„Wie akut ist der geschilderte Fall? 1=nicht akut, 10=sehr akut“ ______
„Wie emotional belastend ist der geschilderte Fall? 1 =nicht belastend, 10 = sehr belastend“ ______

3 Allgemeine Daten für die statistischen Einordung

3.1 Bekanntheit von medizinisch mobilen Ressourcen

„Nun möchte ich Ihnen gerne Fragen zu den Telefonnummern stellen.
Welche Telefonnummern kennen Sie, unter denen man medizinische Hilfe bekommen kann?“

Notruf/Rettungsdienst (SMH)	☐	(☒ = eigenständig genannt)
Wenn nicht eigenständig genannt: *„Kennen Sie den Rettungsdienst?“*	☐ ja	☐ nein
„Wie lautet die Nummer vom Notruf/Rettungsdienst?“	☐	(☒ = konnte Nummer nennen)
„Woher kennen Sie die Notruf-/Rettungsdienstnummer?“	______:______	
Ärztlicher Bereitschaftsdienst (ÄBD,DHD)	☐	(☒ = eigenständig genannt)
Wenn nicht eigenständig genannt: „Kennen Sie den *Ärztlichen Bereitschaftsdienst?“*	☐ ja	☐ nein
„Wie lautet die Nummer vom Ärztlichen Bereitschaftsdienst?“	☐	(☒ = konnte Nummer nennen)
„Woher kennen Sie die Nummer vom Ärztlichen Bereitschaftsdienst?“	______:______	
Weitere: ______		

3.2 Angaben zum Befragten

„Um die Daten wissenschaftlich auswerten zu können, ist es uns wichtig, dass Sie uns abschließend einige allgemeine Fragen beantworten. “

„Welches Geschlecht haben Sie?“	☐ männlich ☐ weiblich ☐ andere ☐ k. A.
„In welchem Jahr sind Sie geboren?“	______ ☐ k. A.
„In welchem Bundesland leben Sie?“	______ ☐ k. A.
„Wie groß ist Ihr Wohnort? Wählen Sie eine der folgenden Möglichkeiten:“	☐ Großstadt<br>☐ Rand oder Vororte einer Großstadt<br>☐ Mittel- oder Kleinstadt

Fragebogen

	☐ ländliches Dorf<br>☐ Einzelgehöft oder allein stehendes Haus auf dem Land<br>☐ k. A.
„Wie ist Ihr Familienstand?“	☐ Verheiratet, zusammenlebend<br>☐ Verheiratet, getrenntlebend<br>☐ ledig<br>☐ geschieden /eingetragene Partnerschaft aufgehoben<br>☐ verwitwet /Lebenspartner/in verstorben<br>☐ eingetragene Partnerschaft, zusammenlebend<br>☐ eingetragene Partnerschaft, getrenntlebend<br>☐ k. A.
„Wie viele Personen leben in Ihren Haushalt?“	______, ______ davon unter 18 Jahre ☐ k. A.
„Wie hoch ist das monatliche Haushaltsnettoeinkommen? Also das Gesamteinkommen aller Personen im Haushalt nach Abzug der Steuern.“	☐ <900€ ☐ 2600-3600€<br>☐ 900-1300€ ☐ 3600-5000€<br>☐ 1300-1500€ ☐ 5000-18000€<br>☐ 1500-2000€ ☐ k. A.<br>☐ 2000-2600€
„Nennen Sie mir Ihren höchsten Bildungsabschluss“	nach allg. Schulausbildung:<br>☐ noch in schulischer Ausbildung<br>☐ Haupt-(Volks-)schulabschluss<br>☐ Abschluss der polytechnischen Oberschule<br>☐ Realschul- oder gleichwertiger Abschluss<br>☐ Fachhochschul- oder Hochschulreife<br>☐ ohne Angabe zur Art des Abschlusses<br>☐ ohne allgemeinen Schulabschluss<br>nach beruflichem Bildungsabschluss:<br>☐ Lehre/Berufsausbildung im dualen System<br>☐ Fachschulabschluss<br>☐ Fachschulabschluss in der ehemaligien DDR<br>☐ Fachhochschulabschluss<br>☐ Hochschulabschluss<br>☐ Bachelor ☐ Master ☐ Diplom<br>☐ Promotion<br>☐ ohne beruflichen Bildungsabschluss
**Migrationshintergrund in engeren Sinne:**<br>*„Haben Sie von Geburt an die deutsche Staatsbürgerschaft?*<br><br>*Haben Ihre Eltern von Geburt an die deutsche Staatsbürgerschaft?*<br><br>Wenn nein: *„Leben Sie mit einem Elternteil, das nicht die deutsche Staatsbürgerschaft hat, zusammen in einem Haushalt?*	☐ ja ☐ k. A.<br>☐ nein, *„aus welchem Land kommen Sie?“* ______<br><br>☐ ja ☐ nein, *„aus welchem Land kommen sie?“* ______<br><br>☐ ja ☐ nein

3.3 Medizinische Vorkenntnisse

1. *„Haben Sie eine medizinische Ausbildung bzw. Studium?“* ☐ ja, welche: ______ ☐ nein ☐ k. A.
2. *„Sind Sie oder waren Sie ehrenamtlich im medizinischen Bereich tätig?“* ☐ ja, als: ______ ☐ nein ☐ k. A.
3. *„Haben Sie privat oder beruflich regelmäßig mit med. Fachpersonal zu tun?“* ☐ ja ☐ nein ☐ k. A.

4 Abschluss

„Vielen Dank, dass Sie sich die Zeit genommen haben und für Ihre freundliche Auskunft. Wir wünschen Ihnen noch einen angenehmen Tag.“
